# Supplementary material for: Differentiation of Bifidobacterium longum subspecies longum and infantis by quantitative PCR using functional gene targets
Source: PeerJ. 2017 May 25;5:e3375. doi: 10.7717/peerj.3375 (PMC5446769; doi:10.7717/peerj.3375)
Supplement: Figure S1 — Effect of baby nutrition (breast milk, cow’s milk formula) on relative abundances of B. longum subsp. infantis in the feces of caesarean-delivered Chinese children. The relative abundance (B. longum subsp. infantis abundance/Total 16S rRNA gene target abundance) data was normalized by log transformation and evaluated statistically by one-way ANOVA with multiple comparisons. Scatter plots with means and SEM are shown. The test limit of detection was 0.01% relative abundance. [file peerj-05-3375-s001.pdf]

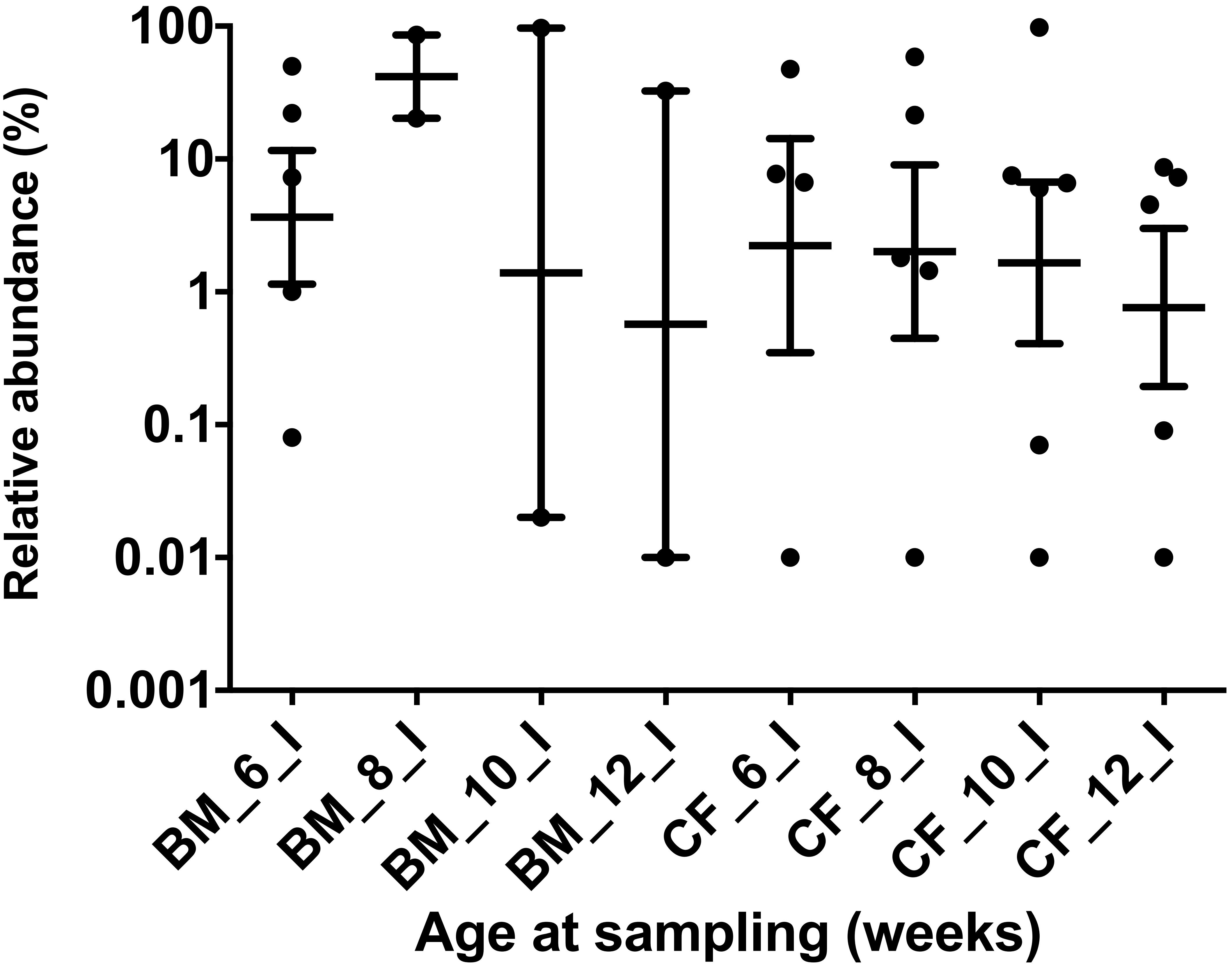

Figure S1. Effect of baby nutrition (breast milk, cow's milk formula) on relative abundances of *B. longum* subsp. *infantis* in the feces of caesarean-delivered Chinese children. The relative abundance (*B. longum* subsp. *infantis* abundance/Total 16S rRNA gene target abundance) data was normalized by log transformation and evaluated statistically by one-way ANOVA with multiple comparisons. Scatter plots with means and SEM are shown. The test limit of detection was 0.01% relative abundance.
